# Gradient local anesthesia for percutaneous endoscopic interlaminar discectomy at the L5/S1 level: a feasibility study

**DOI:** 10.1186/s13018-020-01939-5

**Published:** 2020-09-15

**Authors:** Wan-Li Feng, Jun-Song Yang, Dongmei Wei, Han-Lin Gong, Yong Xi, Hui-Qiang Lv, Xin-Gang Wang, Bin Xia, Jian-Min Wei

**Affiliations:** 1 Department of Spine Surgery, Baoji City Hospital of Traditional Chinese Medicine, Baoji, Shaanxi People’s Republic of China; 2 Department of Spine Surgery, Honghui Hospital, Xi’an Jiaotong University, No. 76 Nanguo Road, Xi’an, 710054 Shaanxi People’s Republic of China; 3 Department of Gynecology and Obstetrics, West China Second University Hospital, Sichuan University, Chengdu, 610041 People’s Republic of China; 4 Department of Integrated Traditional Chinese and Western Medicine, West China Hospital, Sichuan University, Chengdu, 610041 Sichuan People’s Republic of China; 5 Department of Orthopaedics, Tongchuan People’s Hospital, Tongchuan, Shaanxi People’s Republic of China

**Keywords:** L5/S1 disc herniation, Percutaneous endoscopic lumbar discectomy, Interlaminar approach, Local anesthesia, Effect

## Abstract

**Background:**

During the process of shearing the ligamentum flavum, rotating the working channel, and manipulating the annulus fibrosis, the sinuvertebral nerve and the spinal nerve root can be irritated, inducing intolerable back and leg pain. Thus, general anesthesia is recommended and well accepted by most surgeons when performing percutaneous endoscopic lumbar discectomy (PELD) via the interlaminar approach. The aim of our study was to explore the efficacy and safety of percutaneous endoscopy interlaminar lumbar discectomy with gradient local anesthesia (LA) in patients with L5/S1 disc herniation.

**Methods:**

This retrospective study was conducted between December 2017 and June 2018. The study included 50 consecutive patients who met the study criteria, had single-level L5/S1 disc herniation, and underwent PELD via the interlaminar approach under gradient LA. Different concentrations of local anesthetic compound (LAC) were injected into different tissues inside and outside the ligamentum flavum to complete gradient LA. The evaluation criteria included the intraoperative satisfaction score, visual analog scale (VAS) score, Oswestry Disability Index (ODI), complications, and adverse reactions.

**Results:**

The intraoperative satisfaction score was consistently over 7, with an average score of 9.3 ± 0.7, indicating that LAC can achieve satisfactory pain control throughout the PELD operation without additional anesthesia. The postoperative VAS score and ODI were dramatically improved at each follow-up interval (*P* < 0.001, respectively). There was no serious complication such as dural rupture caused by puncture, dural laceration caused by manipulation under endoscopy, total spinal anesthesia, iatrogenic nerve root injury, epidural hematoma, infections, or local anesthetic-related adverse reactions. Three patients experienced transient postoperative dysesthesia of the lower limbs that gradually recovered within 24 h.

**Conclusions:**

Gradient local anesthesia can satisfactorily and safely control intraoperative pain during the PELD via the interlaminar approach. It can not only improve intraoperative satisfaction, but also reduce local anesthesia-related adverse reactions and surgery-related complications.

## Background

Percutaneous endoscopic lumbar discectomy (PELD) is a new, minimally invasive procedure for the treatment of various degenerative lumbar disc diseases. Compared with traditional open surgery, PELD is equally effective in the treatment of lumbar disc herniation [1]. PELD also causes less damage to the paravertebral muscle and lower bleeding loss, and is associated with a shorter hospital stay and faster functional recovery postoperatively [2–4].

PELD is commonly performed using a transforaminal or interlaminar approach [3, 5]. For L5/S1 disc herniation, the transformational route is reported to be technically demanding in terms of anatomic constraints in the lumbosacral region, such as a high iliac crest, thickened L5 transverse process, hypertrophied facet joint, and narrowing foramen [6–9]. The interlaminar distance is greatest at the L5/S1 level, with the average width reported to be up to 31 mm [10]. PELD via the interlaminar approach may be the ideal alternatives to the transformational approach.

During the process of shearing the ligamentum flavum, rotating the working channel, and manipulating the annulus fibrosis, the sinuvertebral nerve and the spinal nerve root can be irritated, inducing intolerable back and leg pain. Thus, general anesthesia (GA) is recommended and well accepted by most surgeons when performing PELD via the interlaminar approach. However, several researchers have confirmed the feasibility of conducting percutaneous endoscopy interlaminar lumbar discectomy with local anesthesia (LA), citing the advantages of a faster recovery, shorter hospital stay, lower hospital cost, and fewer complications, such as postoperative cognitive dysfunction and iatrogenic nerve root injury. However, anesthesia-related complications, including nausea and vomiting, as well as a poor anesthetic effect, are noted in these studies [11–14].

In the present study, we evaluated modifying the proportion and administration of local anesthetics and first propose the technique of gradient LA. The aim of our study was to explore the efficacy and safety of percutaneous endoscopy interlaminar lumbar discectomy with gradient LA in patients with L5/S1 disc herniation.

## Methods

This retrospective study was conducted between December 2017 and June 2018. The study included 50 consecutive patients who met the study criteria, had single-level L5/S1 disc herniation, and underwent PELD via the interlaminar approach under gradient LA. More specifically, the study included 32 male and 18 female patients with an average age of 41.5 ± 6.3 years (range, 24–62 years).

**Fig. 1:**
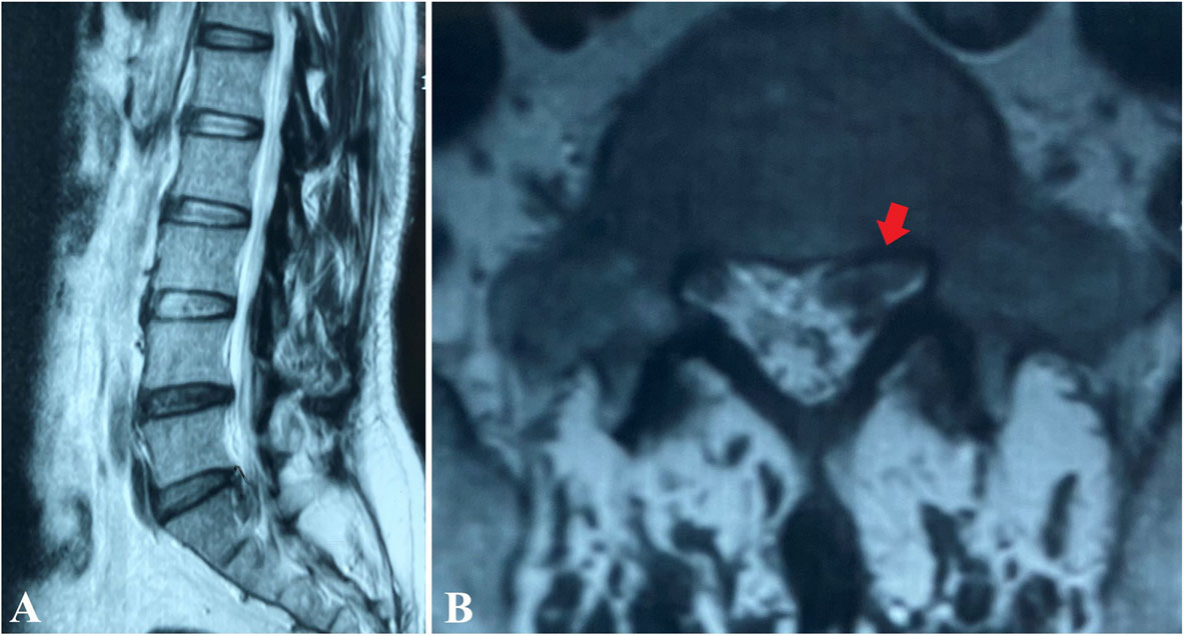
Illustrated case of a 48-year-old female patient. Preoperative T2-weighted MRI of the lumbar spine showed the prolapse of L5/S1 disc in the sagittal section (**a**). The axial plane of MRI showed the left S1 nerve root was compromised (**b**, red arrow)

The inclusion criteria were as follows: typical radiating leg pain with or without back pain, magnetic resonance imaging (MRI) suggesting a single-level lumbar disc herniation at L5/S1 (which could explain neurological symptoms) (Fig. 1), failed conservative treatments for 6 weeks or the neurological deficit progressing, and the patient accepting PELD via the interlaminar approach under gradient LA.

The exclusion criteria were as follows: PELD through the transforaminal approach, refusing to undergo gradient LA, patients with extreme lateral or central type lumbar disc herniation or lumbar spinal stenosis, previous lumbar surgery or interventional treatment at L5/S1, and accompanying kidney or liver dysfunction, hematopoietic or cardiovascular disease, or psychotic disorder not compatible with surgery.

The Medical Ethics Committee of our hospitals approved the study in accordance with the relevant guidelines and regulations. Informed consent was obtained from all patients.

### Surgical equipment

The main surgical instruments included the endoscopic system and bipolar radiofrequency (Spinendos® GmbH, München, Germany), and the endoscopic-matched ultrasonic osteotome device (XD880A, SMTP, Beijing, China).

### Gradient local anesthesia

Surgeries were conducted by three experienced spinal surgeons who had carried out 200 PELD procedures. We modified the procedure of conventional LA and developed two-step gradient strategies for local anesthesia. With the ligamentum flavum as the boundary, the different anesthetic compound was utilized.

**Fig. 2:**
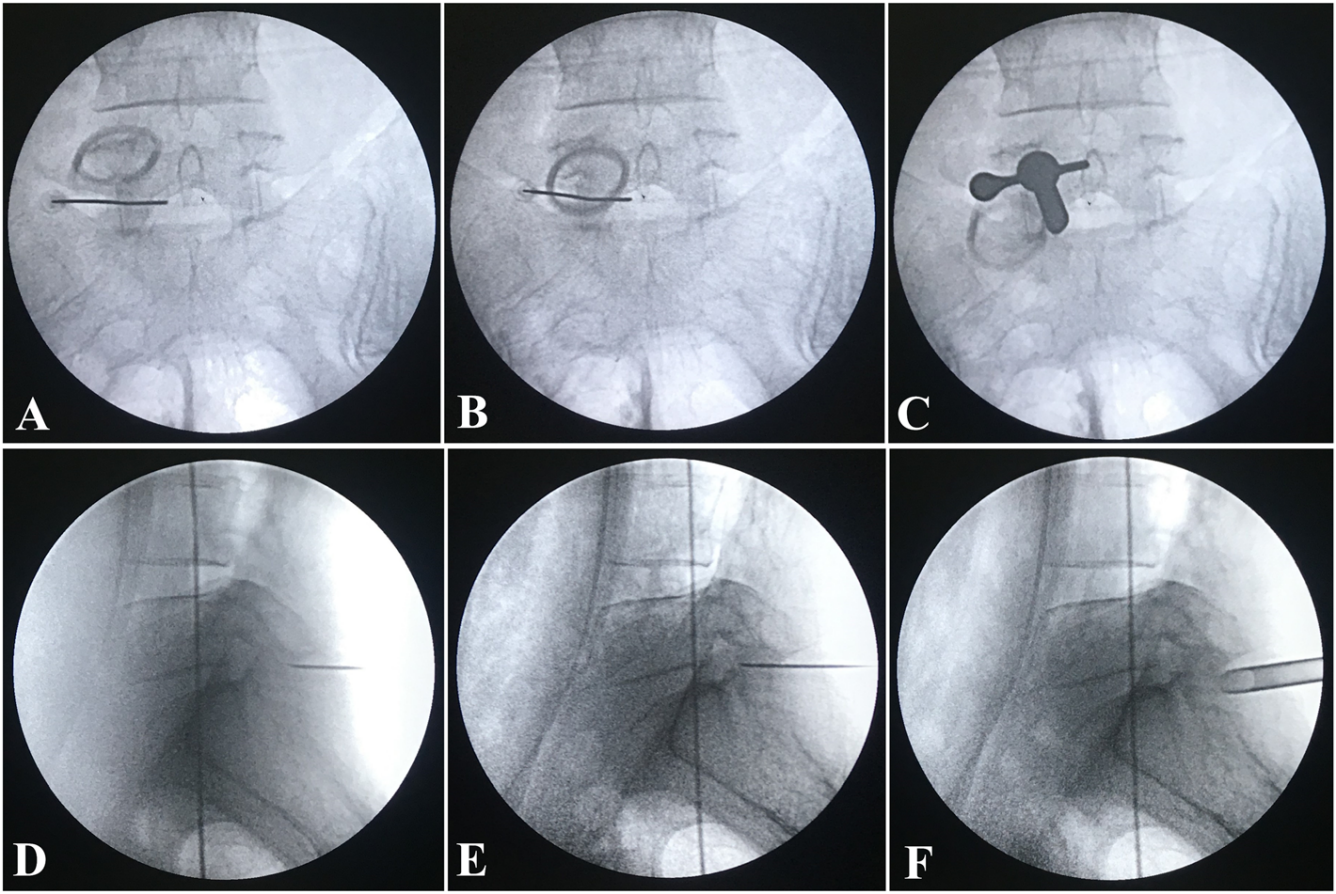
A 22-gauge spinal needle was used to complete the local anesthetic administration for the area behind the ligamentum flavum, which was confirmed at the lateral (**a**) and anterioposterior (**d**) plane. The spinal needle was placed at surface of the central area of the interlaminar space of L5/S1 along with the spinous process and then advanced until the sensation of loss-of-resistance occurred, which was confirmed at the lateral (**b**) and anterioposterior (**e**) plane. After gradient LA, routine PELD via the interlaminar approach was performed. The working channel was established, which was confirmed at the lateral (**c**) and anterioposterior (**f**) plane

**Fig. 3:**
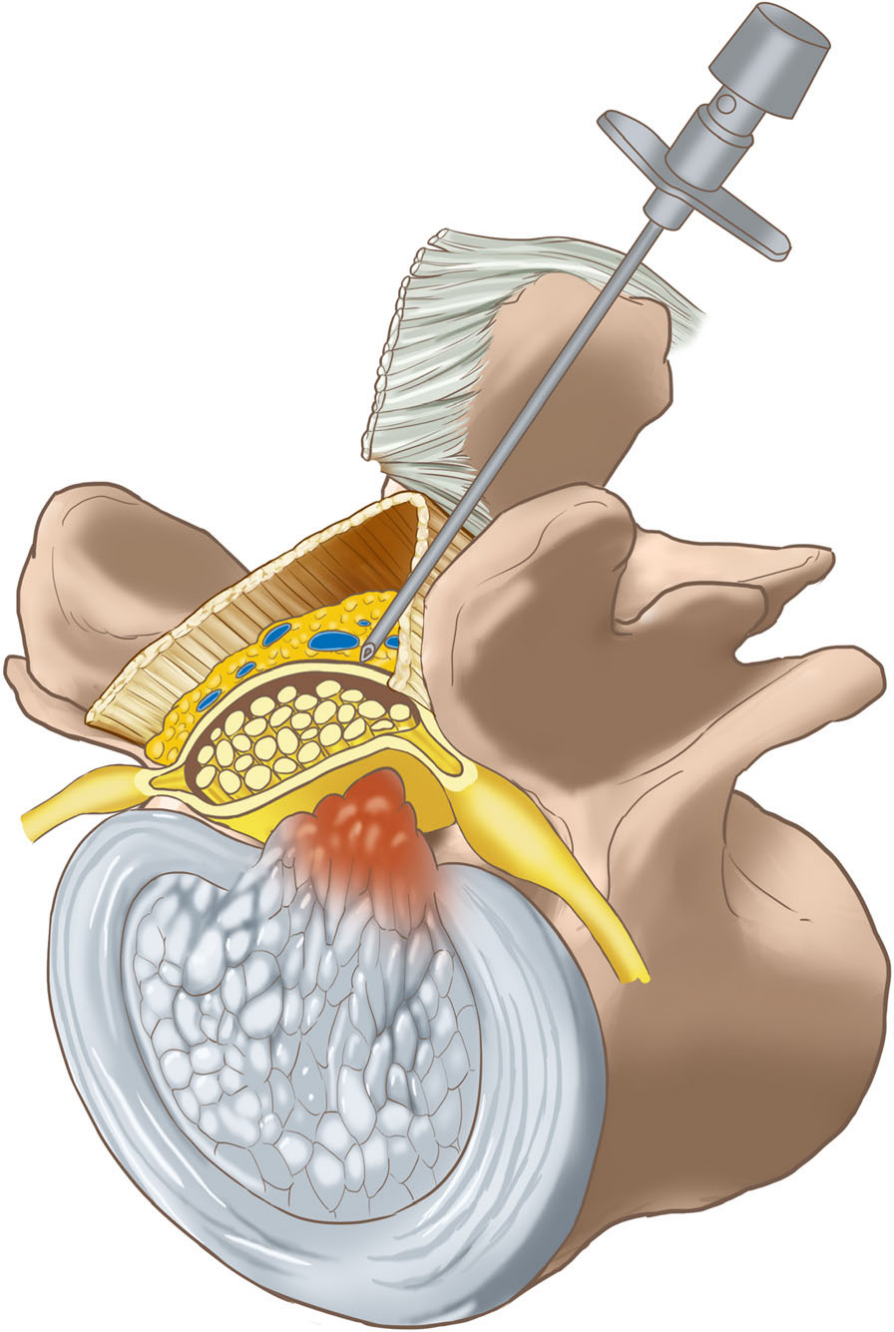
The schematic diagram showed the spinal needle entered the posterior epidural space

The local anesthetic compound (LAC) consisted of 10 mL of 2% lidocaine, 10 mL of 1% ropivacaine, and 20 mL of 0.9% saline solution. Firstly, a 22-gauge spinal needle was used to complete the local anesthetic administration for the area behind the ligamentum flavum (Fig. 2a, d). During the procedure, 5 mL of LAC infiltrated into the skin and subcutaneous soft tissues, 15 mL of LAC covered the periosteum of the cranial and caudal laminae and the medial portion of the ipsilateral facet joint, and 5 mL of LAC was injected into the superficial layer of the ligamentum flavum. Secondly, the spinal needle was placed at the surface of the central area of the interlaminar space of L5/S1 along with the spinous process and then advanced until the sensation of loss-of-resistance occurred. Negative pressure during suction and no fluid entering the syringe confirm that the spinal needle entered the posterior epidural space (Figs. 2b, e and 3). In addition to 5 mL of LAC, 5 mL of 2% lidocaine was also injected into the posterior epidural space to enhance the local anesthetic effect.

**Fig. 4:**
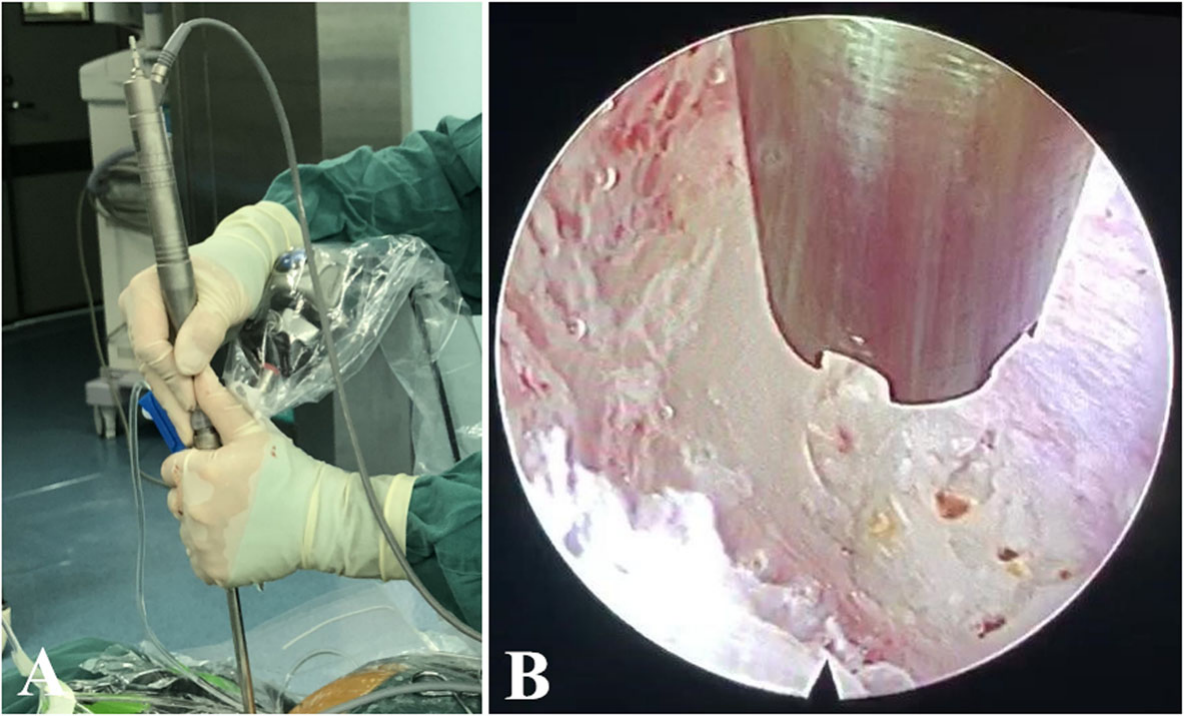
The partial lamina was removed safely and efficaciously with the assistance of the ultrasonic osteotome (**a**) under the monitoring of endoscope (**b**)

**Fig. 5:**
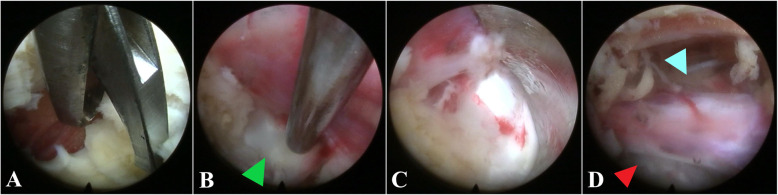
When the ligamentum flavum was opened with basket forceps (**a**), the herniated disc was visible (**b**). Rotating the working channel and pushing the S1 nerve root away from the working channel (**c**), the intervertebral disc tissue can be excised to achieve the ideal decompression of S1 nerve on the shoulder (red arrow head) and axillar (blue arrow head) region (**d**)

After gradient LA, routine PELD via the interlaminar approach was performed. The partial lamina and the inferior articular process were removed safely and efficaciously with the assistance of the ultrasonic osteotome (Fig. 4). The working channel was rotated painlessly to push the nerve root gently away from the channel, and the protruded nucleus pulposus was exposed under endoscopic visualization (Figs. 2 and 5c, f). The pulsation of the traversing nerve root can be observed after the complete removal of the protruded nucleus pulposus.

### Evaluation criteria

Besides the operative duration, an intraoperative satisfaction score (between 0 and 10) was introduced to evaluate surgical tolerance and the effect of the intraoperative gradient LA. A score of 7–10 indicates that there was no pain or completely acceptable pain; a score of 4–6 refers to pain of short duration and tolerable pain; a score of 0–3 indicates severe and unendurable pain. The conventional treatments, such as the physical therapy, were only provided to the patients with the severe leg pain (visual analog scale (VAS) score > 4) during follow-up period. Postoperative clinical outcome evaluations were assessed at 1 week, 1 month, 3 months, and 6 months postoperatively, including a VAS score between 0 and 10 representing the severity of leg pain (with 0 representing no pain and 10 representing the highest degree of pain) and the Oswestry Disability Index (ODI). Complications and adverse reactions within 24 h after surgery such as nausea, vomiting, dizziness, and drowsiness are also recorded.

### Statistical analysis

All statistical analyses were performed via GraphPad prism 8.0.2 software (GraphPad, San Diego, CA, USA). Data are presented as means ± standard deviations. The paired *t* test was used to compare the pre- and postoperative VAS score and ODI. A *P* value < 0.05 was considered statistically significant.

## Results

PELD via the interlaminar approach was successfully performed in all 50 patients under gradient LA, and no case transferred to GA intraoperatively. All patients fulfilled at least the 6-month follow-up.

The baseline characteristics of all patients are summarized in Table 1. The average operative time was 50.3 ± 10.3 min. The intraoperative satisfaction score was consistently over 7, with an average score of 9.3 ± 0.7, indicating that LAC can achieve satisfactory pain control throughout the PELD operation without additional anesthesia.

**Table 1:** General information for patients who underwent PELD via the interlaminar approach under the gradient local anesthesia (*x̄* ± *s*)

Demographics	
Male/female	32/18
Mean (range), years	41.5 ± 6.3
Body weight (kg)	64.2 ± 5.4
Types of disc herniation	
Paracentral	31
Prolapses/sequestered	19

*PELD* percutaneous endoscopy lumbar discectomy

As shown in Table 2, the postoperative VAS score and ODI were dramatically improved at each follow-up interval (*P* < 0.001, respectively). None of the patients received physical therapy during the follow-up period. There was no serious complication such as dural rupture caused by puncture, dural laceration caused by manipulation under endoscopy, total spinal anesthesia, iatrogenic nerve root injury, epidural hematoma, or infections. In terms of local anesthetic-related adverse reactions, no patient complained of nausea, vomiting, dizziness, or drowsiness within 24 h postoperatively. Three patients experienced transient postoperative dysesthesia of the lower limbs that gradually recovered within 24 h.

**Table 2:** Comparison of the VAS score and ODI of patients before surgery, 1 week, and 1, 3, and 6 months postoperatively (*x̄* ± *s*)

	Preoperatively	1 week postoperatively	1 month postoperatively	3 months postoperatively	6 months postoperatively
VAS score	7.3 ± 1.1	2.6 ± 0.7^a^	2.2 ± 0.4^a^	1.9 ± 0.4^a^	1.5 ± 0.5^a^
ODI	48.2 ± 6.8	32.6 ± 8.3^a^	24.3 ± 7.7^a^	18.4 ± 8.1^a^	14.3 ± 6.7^a^

*VAS* visual analog scale, *ODI* Oswestry Disability Index

^a^Compared to preoperatively *P* < 0.001

## Discussion

With the improvement of surgical techniques and equipment, PELD has become one of the most common minimally invasive surgeries in spinal surgery practice. Since most surgeons are accustomed to performing posterior spine surgery and are familiar with the anatomy of the posterior spine, PELD via the interlaminar approach is more acceptable to most surgeons than the transforaminal approach.

For lumbar disc herniation at L5/S1, the interlaminar approach takes advantage of a natural anatomical feature (a relatively large interlaminar space) and provides minimally invasive and targeted decompression for the prolapse or sequestration of the nucleus pulposus in the spinal canal. Compared to the transforaminal approach, PELD via the interlaminar approach resulted in greater retraction and manipulation to the dural matter and nerve, which can cause uncomfortable pain and hinder the operation. Thus, most surgeons prefer to perform the endoscopic discectomy through the interlaminar approach under GA. However, GA requires preoperative fasting and is associated with slower recovery and more medical expense.

Several authors have compared the effect of GA and LA on the clinical outcome of PELD via the interlaminar approach. Ye et al. included 60 patients with lumbar disc hernia who were treated with PELD with an interlaminar approach [11]. At each follow-up of 3, 6, and 12 months after surgery, there was no significant difference in ODI and VAS scores between GA and LA. However, 1 patient in the GA group had intraoperative nerve root injury. There were 2 cases of adverse reactions in the LA group and 6 such cases in the GA group. From the perspective of the intraoperative anesthesia effect, compared with the 100% satisfaction rate of the GA group, only 50% of the patients in the LA group were satisfied with the anesthesia effect. Another study performed by Chen et al. included 123 patients with L5/S1 disc herniation [12]. They also observed a similar improvement of neurological function; however, one patient developed dural laceration and nerve root injury intraoperatively in the GA group. Transient sensory disturbance was observed in 22 patients (12 in the GA group and 10 in the LA group). Thus, the authors concluded that LA is superior to GA for PELD via the interlaminar approach. Notably, they also found that, although patients in the LA group tolerated the procedure, they experienced varying degrees of discomfort during the operation. This led to further modification in the technique for LA by the two authors. Guan et al. injected the lidocaine into the epidural space with a long needle under endoscopic monitoring [13]. Among the reported 120 patients, only one patient developed postoperative cognitive dysfunction (within the GA group). A lower intraoperative VAS score and higher satisfaction reflected the advantages of LA over GA. Unfortunately, however, this study does not mention local anesthetic-related adverse reactions or surgery-related complications, making it difficult to accurately assess the safety of PELD under LA.

Wu et al. proposed a technique called stepwise local anesthesia that consists of conventional local anesthesia, epidural injection, and nerve root block procedures, and reported that the anesthetic effect was excellent/good in 97.9% (47 patients) of patients of the stepwise local anesthesia group [14]. Nine patients experienced complications associated with local anesthesia, including dyspnea, temporary paresis of the legs, and temporary worsened dysesthesia or numbness in the legs.

Except for Chen et al. [12], who did not elaborate on specific anesthetic drugs, the other three studies reported the specific composition of the LA anesthetic drug [11, 13, 14]. The duration of anesthesia with a single dose of lidocaine may not be long enough for PELD. Instead of using lidocaine alone [13], a mixture of ropivacaine and lidocaine was applied in the Ye et al. and Wu et al. studies [11, 14]. We also added ropivacaine with a longer half-life to enhance the anesthetic efficacy and extend the duration of anesthesia. Additionally, the advantage of ropivacaine is that a low concentration of ropivacaine can perform selective sensory blockade and maintain the motor function of neurons, so that nerve function can be monitored in real time through intraoperative lower limb movement [15].

We disagree with Wu et al.’s reference to the technique of nerve root block, during which the needle tip is penetrated into the nerve root under endoscopic view [14]. Intraneural injections can accelerate the occurrence of nerve block, increase the success rate, and prolong block time [16, 17]. However, it has also been reported that intraneural injections may cause iatrogenic neurological injury and functional deficits [18–20].

Wu et al. recommended that, if LA is not satisfactory, nerve root injection can be repeated until the pain is satisfactorily controlled [14]. It also suggests that the previous two-step anesthesia may not be as effective. Moreover, repeated administration of the anesthetic compound is the main cause of overdosage, thus increasing the possibility of anesthetic-related adverse reactions. In our modified formula for an anesthetic drug compound, the saline solution is reduced from 30 to 20 mL and the anesthetic concentration is increased. The effect of local anesthesia could be enhanced temporarily by injecting 5 mL of lidocaine before the ligamentum flavum is opened. Otherwise, the injected drug will be quickly washed away by the irrigating water, leading to sub-anesthetic results. The total amount of anesthetic is within a safe dose, which greatly reduces the adverse reactions associated with local anesthetics. That explains why there were no adverse reactions in our case series. Although the drug was injected into the epidural space without endoscopic monitoring, dural rupture caused by puncture did not occur because the puncture point was chosen at the central area of the interlaminar space of L5/S1, close to the spinous process. Anatomically, the widest part of the epidural space is in the midline below the spinous process, known as the posterior epidural space. When the disc is protruded, the epidural space plays a compensatory role, reducing the mechanical compression of the nerve root. However, the posterior epidural space is usually the last to be compromised. Thus, epidural administration in this area is quite safe and there is no intraoperative dural puncture injury or occurrence of total spinal anesthesia.

In terms of the efficacy of intraoperative anesthesia, all patients had intraoperative satisfaction scores greater than 7 points, indicating that LAC induces satisfactory pain control throughout the whole surgery. Based on the ideal anesthetic effect, all patients completed thorough neural decompression and achieved significant improvement in symptoms compared with their preoperative symptoms.

The main limitation of our study is the retrospective non-randomized controlled study design. Further studies with a high level of evidence are needed to compare the benefits and safety of gradient local anesthesia with GA for PELD via the interlaminar approach.

## Conclusions

Gradient local anesthesia can satisfactorily and safely control intraoperative pain during the PELD via the interlaminar approach. It can not only improve intraoperative satisfaction, but also reduce local anesthesia-related adverse reactions and surgery-related complications. This is a promising alternative to anesthesia for some patients, especially elderly patients with multiple diseases who cannot tolerate general anesthesia.

## Data Availability

The datasets used and/or analyzed during the current study are available from the corresponding author on reasonable request.

## Abbreviations

PELD: Percutaneous endoscopic lumbar discectomy
GA: General anesthesia
LA: Local anesthesia
MRI: Magnetic resonance imaging
LAC: Local anesthetic compound
VAS: Visual analog scale
ODI: Oswestry Disability Index
